# Metal-based nanodrugs for cancer immunotherapy: smart nanosystems, immunomodulatory mechanisms, and translational perspectives

**DOI:** 10.3389/fchem.2026.1911801

**Published:** 2026-07-29

**Authors:** Shu Yan, Fei Zhou

**Affiliations:** 1 Department of Tumor Radiotherapy, Jilin City Hospital of Chemical Industry, Jilin, China; 2 Medical Image Center, Jilin Central Hospital, Jilin, China

**Keywords:** cancer immunotherapy, metal-based nanodrugs, metalloimmunology, STING pathway, tumor microenvironment

## Abstract

The clinical benefits of cancer immunotherapy are primarily limited by low response rates and an immunosuppressive tumor microenvironment (TME). Metal elements such as iron, manganese, copper, and zinc, as essential micronutrients for the body, play a key regulatory role in the development and effector functions of immune cells, giving rise to the interdisciplinary field of metalloimmunology. However, the poor tumor-targeting ability of free metal ions, their high systemic toxicity, and rapid *in vivo* metabolism severely hinder their clinical translation. This review systematically summarizes research progress on metal nanomedicines as smart delivery systems in tumor immunotherapy, outlining design paradigms for nanoplatforms from an engineering perspective, covering microenvironment-responsive release, active targeting strategies, and multifunctional integration concepts. It elucidates the molecular mechanisms by which metal ions regulate antitumor immunity by inducing immunogenic cell death (ICD), activating the stimulator of interferon genes (STING) signaling pathway, and reshaping the metabolic microenvironment, and explores synergistic strategies combining metal nanomedicines with immune checkpoint blockade (ICB), photothermal therapy (PTT), or photodynamic therapy (PDT). Unlike previous reviews that often focus on either the immunological functions of metal ions or the engineering of nanocarriers in isolation, this review provides an integrated framework that explicitly links metal ion identity, immune pathway engagement, nanoplatform design parameters, and translational barriers. Central to this framework is a mechanism-design matching principle that connects the specific temporal and intensity requirements of each immune activation pathway with tailored material selection and release kinetics. These immune effects are context-dependent and may vary in selected preclinical models. This paper integrates metallobiology, nanomedicine, and tumor immunology to provide a theoretical framework for the precise design of smart metal immunotherapeutics, while identifying key challenges for clinical translation, such as the therapeutic safety window and metal homeostasis compensation mechanisms.

## 1 Introduction

Cancer immunotherapy, particularly the use of immune checkpoint inhibitors (ICIs), has achieved durable clinical responses in various solid tumors (Huang et al., 2025; Mc Neil and Lee, 2025). However, the objective response rates for ICIs generally range from 10% to 40%, primarily due to the complexity of tumor immune evasion (Relecom et al., 2021). Cold tumors, which lack immune cell infiltration, are inherently resistant to immunotherapy. Even when infiltration is present, the accumulation of regulatory T cells, M2 macrophages, and myeloid-derived suppressor cells within the TME can lead to the exhaustion of effector T cells (Kong et al., 2025; Li and Wang, 2026). Therefore, reshaping the TME to transform cold tumors into an immuno-inflammatory phenotype remains a central scientific challenge for overcoming the current bottlenecks in immunotherapy (Huang et al., 2026). Recent studies have revealed that metal ions such as manganese, iron, and copper can regulate antitumor immunity by activating the STING pathway or inducing ICD (Ge et al., 2021; Liang et al., 2021a; Shi et al., 2021; Tang et al., 2020; Wang et al., 2021; Zhang et al., 2023), giving rise to the interdisciplinary field of metalloimmunology. However, free metal ions exhibit poor tumor selectivity, high systemic toxicity, and rapid *in vivo* metabolism, making them unsuitable for direct use in systemic therapy (Darwesh et al., 2023; Lynes et al., 2007; Young et al., 2019; Zhao et al., 2022). Taking iron as an example, although free ferrous ions can generate reactive oxygen species (ROS) via the Fenton reaction, they lack tumor selectivity (Liang et al., 2021b). Nano-delivery systems provide effective tools to address this challenge. By encapsulating metal ions within inorganic nanoparticles, metal-organic frameworks (MOFs), or single-atom nanoenzymes, these systems can achieve passive targeting via the enhanced permeability and retention (EPR) effect, acquire active targeting capabilities through surface modification, and release metal ions on demand in response to the TME’s slightly acidic pH, high glutathione (GSH) levels, or ROS (Bandyopadhyay et al., 2023; Suliman et al., 2023). This on-demand controlled release provides unprecedented tools for precise regulation of metal-based immunotherapy, and such spatiotemporal precision is key to unlocking its full potential (Balwe et al., 2024; Chen et al., 2024; Yang et al., 2023).

Although several recent reviews have addressed aspects of metal-based immunotherapy, their scope and focus remain relatively narrow. For instance, Xia et al. (2025) systematically reviewed copper-based nanomedicines in activating tumor immunity through oxidative stress modulation, providing valuable insights into copper-specific mechanisms and regulated cell death pathways. However, most existing reviews concentrate on a single metal element, a specific nanoplatform type, or isolated immune pathways, without offering an integrated framework that bridges metal ion identity, immune pathway engagement, nanoplatform engineering, and translational barriers. This review focuses on recent advances in the use of metal nanomedicines in cancer immunotherapy. We first outline the immunoregulatory mechanisms of key metal ions such as manganese, iron, copper, and zinc, with particular attention to the differences in kinetics and intensity with which these ions activate immune pathways. We then discuss design strategies for smart nanoplatforms from an engineering perspective and propose a matching framework of mechanism and design to guide material selection and optimization of release behavior. We next explore the synergistic application of metal nanomedicines with PTT/PDT, radiotherapy, and immunotherapy. Finally, we assess the current status of clinical translation, safety challenges, and future directions. By systematically integrating metallobiology, nanomedicine engineering, and tumor immunology across four key metal ions, and by incorporating underdiscussed translational roadblocks including scalable manufacturing, metal homeostasis compensation, and regulatory science into a cohesive discussion, this review distinguishes itself from existing literature and provides a more comprehensive and actionable roadmap for the clinical development of metal-based immunotherapeutics. Integrating metallobiology, nanomedicine, and tumor immunology, this review provides a theoretical framework for the precise design of smart metal immunotherapeutics.

## 2 Immunomodulatory mechanisms of metal ions and smart nanodelivery design

**FIGURE 1:**
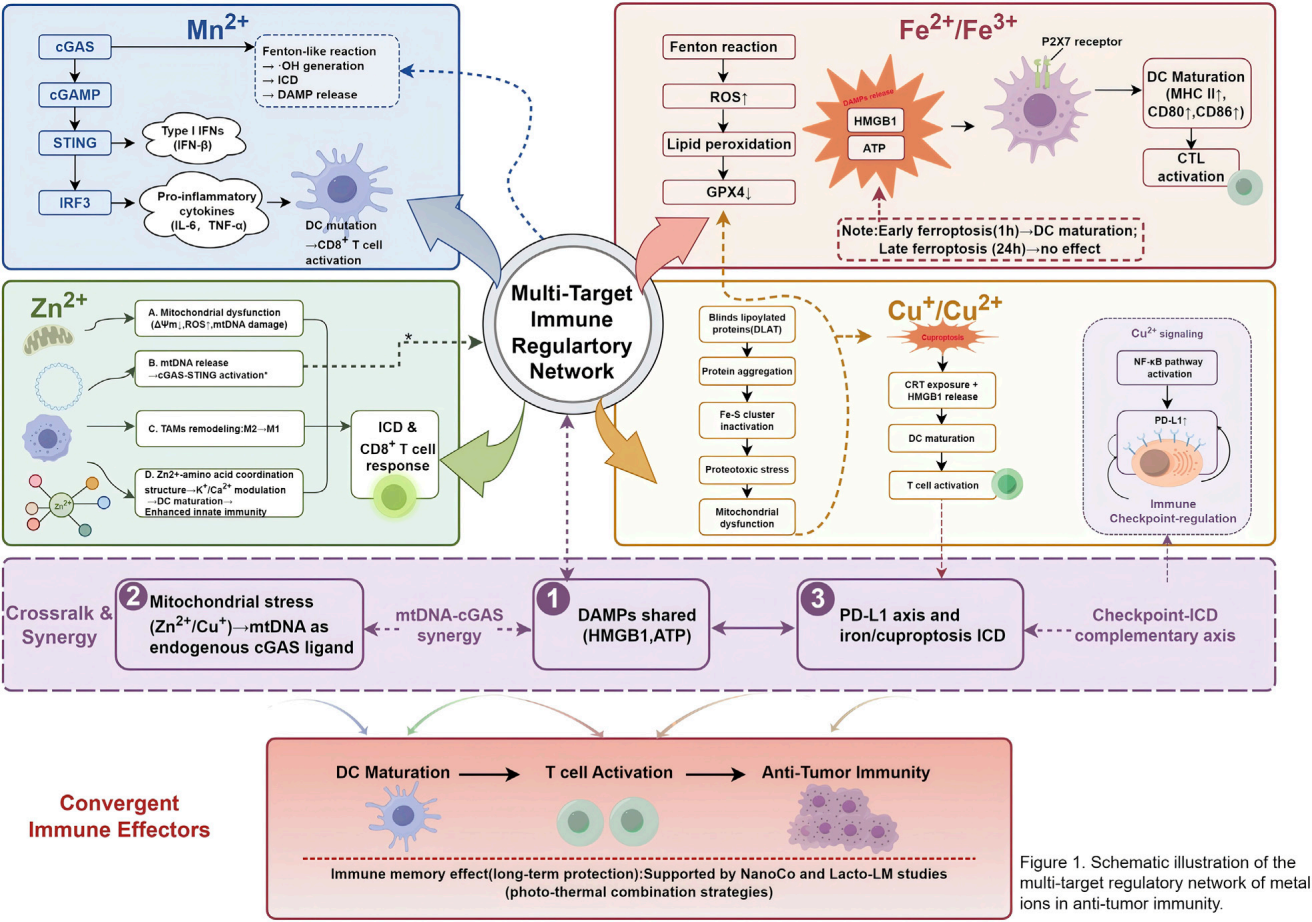
Multi-Target Regulatory Network of Metal Ions in Anti-Tumor Immunity. This four-part schematic illustrates the immune pathways activated by Mn^2+^, Fe^2+^/Fe^3+^, Cu^+^/Cu^2+^, and Zn^2+^. Mn^2+^ activates cGAS-STING-IRF3 signaling, inducing type I IFNs and cytokines for DC maturation and CD8^+^ T-cell activation. Fe^2+^/Fe^3+^ drives Fenton reaction-mediated ferroptosis and DAMP release (HMGB1, ATP), activating DCs via P2X7 receptors. Cu^+^/Cu^2+^ triggers cuproptosis via DLAT and mitochondrial dysfunction, alongside NF-κB-mediated PD-L1 upregulation. Zn^2+^ induces mitochondrial stress, mtDNA release for cGAS-STING synergy, TAM M2→M1 polarization, and DC maturation. Dashed connectors indicate crosstalk: shared DAMPs (Fe/Cu), mtDNA-cGAS synergy (Zn/Mn), and PD-L1-ICD complementarity (Cu/Fe). All pathways converge on DC maturation, T cell activation, and anti-tumor immunity. Abbreviations: ATP, adenosine triphosphate; cGAMP, cyclic GMP-AMP; cGAS, cyclic GMP-AMP synthase; CRT, calreticulin; CTL, cytotoxic T lymphocyte; DAMP, damage-associated molecular pattern; DC, dendritic cell; DLAT, dihydrolipoamide S-acetyltransferase; GPX4, glutathione peroxidase 4; HMGB1, high mobility group box 1; ICD, immunogenic cell death; IFN, interferon; IL, interleukin; IRF3, interferon regulatory factor 3; mtDNA, mitochondrial DNA; NF-κB, nuclear factor kappa B; PD-L1, programmed death-ligand 1; ROS, reactive oxygen species; STING, stimulator of interferon genes; TAM, tumor-associated macrophage.

Metal ions, acting as cofactors or signaling molecules, are extensively involved in the development, activation, and effector function of immune cells (Shen et al., 2023). As shown in Figure 1, recent studies have revealed that metal ions such as manganese, iron, copper, and zinc can intervene at multiple key nodes of the tumor immune response, forming a multitarget, interconnected regulatory network (Voli et al., 2020; Xu et al., 2024; Zhang et al., 2024a). Understanding the commonalities and differences in the mechanisms among these ions is essential for the design of smart nanodelivery systems.

### 2.1 Immunomodulatory mechanisms of key metal ions

Regarding the activation of innate immune pathways, both manganese and zinc ions have been found to activate the cyclic GMP-AMP synthase (cGAS)-STING pathway, but their mechanisms of action differ significantly (Dvorkin et al., 2024). Manganese ions act directly as a second messenger for cGAS, enhancing its sensitivity to double-stranded DNA. This catalyzes the production of cyclic GMP-AMP (cGAMP), which activates STING and induces type I interferons and pro-inflammatory cytokines in selected preclinical models (Chen H. et al., 2025; Liang et al., 2024; Wu Z. et al., 2025). This mechanism makes manganese ions a direct STING agonist. In contrast, the pathway by which zinc ions activate STING is more indirect: by inducing mitochondrial dysfunction and oxidative mitochondrial DNA (mtDNA) damage in a dose-dependent fashion, they cause mtDNA to leak into the cytoplasm, thereby indirectly activating the cGAS-STING pathway (Wang Z. et al., 2025; Zou et al., 2026). This damage-release-recognition cascade not only activates innate immunity but also simultaneously triggers ICD, coupling innate immune activation with the initiation of adaptive immunity. The kinetics and intensity of STING activation differ between the two ions: manganese ions induce faster and more direct signaling, whereas zinc ions are associated with more pronounced mitochondrial stress. This distinction suggests that in nanodrug design, manganese-based platforms may be preferable when seeking a rapid and intense type I interferon response; conversely, zinc ions offer unique advantages when aiming to combine immune activation with mitochondrial damage-mediated ICD. However, the activation of the cGAS-STING pathway has a double-edged effect. Moderate activation promotes antitumor immunity in the TME, whereas excessive or sustained activation may produce the opposite effect (Fu et al., 2024). Of particular concern, chronic STING activation driven by chromosomal instability has been shown to promote a pro-metastatic TME, and STING inhibitors reduced metastasis in preclinical models (Li et al., 2023). Therefore, the design of metal nanomedicines must incorporate stimuli-responsive release mechanisms to achieve moderate and transient regulation of STING signaling.

In terms of regulating cell death pathways, iron ions and copper ions induce ferroptosis and cuproptosis, respectively. Both forms of regulated cell death have been shown to be immunogenic; they differ in the intensity of immune activation, the time window, and the accompanying spectrum of danger signals. Ferrous ions generate excessive ROS through the Fenton reaction, leading to the accumulation of lipid peroxides and ultimately resulting in ferroptosis in selected preclinical models (Cao et al., 2026; Delgado-Martín and Martínez-Ruiz, 2024). The immunogenicity of this process exhibits clear time dependence: within 1 h of inducing ferroptosis in certain tumor cell lines, cells release high mobility group box 1 (HMGB1) and ATP, effectively promoting dendritic cell (DC) maturation, whereas these effects are lost after 24 h (Catanzaro et al., 2024; Efimova et al., 2020). This brief time window requires nanomedicines to achieve rapid, high-concentration burst release of ferrous ions at the tumor site; otherwise, the immunostimulatory effect will be significantly diminished. Copper ions, in contrast, induce cuproptosis by directly binding to acylated proteins in the tricarboxylic acid (TCA) cycle, such as dihydrolipoamide S-acetyltransferase (DLAT), leading to abnormal protein aggregation and mitochondrial dysfunction (Tsvetkov et al., 2022). Unlike ferroptosis, cuproptosis does not rely on ROS burst, but its interference with mitochondrial metabolism is more direct. Furthermore, copper ions can upregulate programmed death-ligand 1 (PD-L1) expression in tumor cells via the NF-κB pathway, although this effect is context-dependent and varies with tumor type, copper concentration, and treatment schedule (Li et al., 2025a; Xiong et al., 2023; Zhang W. et al., 2024; Zhang et al., 2025). This finding directly links copper homeostasis to PD-L1-mediated immune evasion, suggesting that the immune-activating effects of copper-based nanomedicines may be offset by PD-L1 upregulation, making combination therapy with ICIs particularly necessary. However, recent *in vivo* evidence indicates that copper delivery can paradoxically suppress antitumor immunity in certain tumor types. With prolonged dosing leading to immune suppression rather than enhancement (Heroux et al., 2026), underscoring the need for careful optimization of dosing schedules.

In addition to directly affecting tumor cells, zinc ions play a unique role in the polarization and functional regulation of immune cells. Zinc ions can promote the polarization of tumor-associated macrophages (TAMs) from the M2 pro-tumor phenotype to the M1 anti-tumor phenotype (Zhang et al., 2024b), an effect that is relatively rare among other metal ions. The coordination complexes formed between zinc ions and amino acids promote DC maturation by regulating intracellular potassium and calcium ion concentrations (Tan et al., 2024), linking trace element metabolism to the functional activation of antigen-presenting cells. Notably, zinc ions exert a dose-dependent double-edged effect on CD8^+^ T cells. At appropriate concentrations, they enhance T-cell activation and cytokine secretion, whereas excessively high concentrations may induce oxidative stress and T-cell apoptosis (Haase and Rink, 2014). This narrow therapeutic window imposes stringent requirements on the release kinetics of nanodelivery systems.

The mechanisms described above are not mutually exclusive. Both manganese and zinc ions can activate the STING pathway, but the former does so directly, whereas the latter does so indirectly; both iron and copper ions can induce ICD, but the accompanying death pathways and profiles of released danger signals differ. Both zinc and copper ions can affect PD-L1 expression. Copper ions upregulate PD-L1, whereas the effect of zinc ions remains unclear. These interactions suggest that combining different metal ions may produce synergistic or antagonistic effects. Therefore, when designing metal nanomedicines, the appropriate metal ion type, combination, and controlled-release delivery strategy should be selected based on the intended immune pathways, the required kinetics, and the need to avoid toxic side effects. Calcium and magnesium ions are also involved in immune regulation; for example, calcium overload can induce mitochondrial damage and pyroptosis (Wang C. et al., 2025), while magnesium promotes metabolic reprogramming of CD8^+^ T cells (Ashique et al., 2023). However, evidence for their direct application in tumor immunotherapy is not yet as robust as that for the four ions mentioned above. Table 1 summarizes the primary immune regulatory functions of representative metal elements, providing a basis for subsequent nanoplatform design.

**TABLE 1:** Immunomodulatory functions of representative metal elements.

Metal element	Major immunomodulatory mechanisms	Representative nanoplatforms/strategies	References
Fe	Fe^2+^/Fe^3+^ induce ferroptosis via Fenton reaction, trigger ICD, release HMGB1 and ATP, and promote DC maturation and tumor-specific T-cell responses.	Nano-carbon iron suspension injection (Phase II clinical trial)	Yang et al. (2025)
Cu	Cu^+^/Cu^2+^ induce cuproptosis by targeting lipoylated proteins of the TCA cycle, and release DAMPs to activate antitumor immunity.	Copper-based MOFs; copper-peptide self-assembled nanoparticles	Li et al. (2025b)
Mn	Mn^2+^ acts as a second messenger of cGAS to activate the STING pathway, induce type I interferon production, promote DC maturation and T cell priming, and convert cold TME into hot TME.	MnO_2_ nanoparticles; Mn^2+^-loaded liposomes; Mn-based MOFs	Chen N. et al. (2025)
Zn	Zn^2+^ induces mitochondrial dysfunction and triggers ICD; polarizes TAMs toward the M1 antitumor phenotype; modulates CD8^+^ T cell activity and metabolism; enhances antigen presentation and cross-priming.	ZIF-8 (Zn-MOF)	Zhou et al. (2025)
Ca	Ca^2+^ overload induces mitochondrial calcium overload and oxidative stress, activates caspase-3/GSDME-mediated pyroptosis, triggers ICD, and promotes TAM polarization toward the M1 phenotype.	CaCO_3_ nanoparticles; CaP nanoplatforms	Qi et al. (2025)
Mg	Mg^2+^ promotes CD8^+^ T cell TCR signaling and metabolic reprogramming, enhances their antitumor activity; synergizes with Mn^2+^ to activate the STING pathway, and promotes DC maturation and long-term immunological memory.	Mg^2+^/Mn^2+^ co-delivery system (e.g., MgCO_3_/MnO_2_ nanoparticles)	Wu J. et al. (2025)
Co	Co^2+^ generates ROS via Fenton-like reactions, induces ICD, and activates the cGAS-STING pathway.	CoF_2_ nanocatalysts (ultrasound-responsive)	Yu et al. (2025)
Gd	Gd^3+^ serves as a T1 MRI contrast agent. Gd-MOF nanosystems induce ICD under microwave hyperthermia, reshape the tumor immune microenvironment, and synergize with microwave hyperthermia and ICB to enhance antitumor immunity.	Gd-MOF	Cui et al. (2024)

Abbreviations: MOFs, metal-organic frameworks; ICD, immunogenic cell death; HMGB1, high mobility group box 1; ATP, adenosine triphosphate; DC, dendritic cell; TCA, tricarboxylic acid; DAMPs, damage-associated molecular patterns; cGAS, cyclic GMP-AMP, synthase; STING, stimulator of interferon genes; TAMs, tumor-associated macrophages; TCR, T cell receptor; MRI, magnetic resonance imaging; ICB, immune checkpoint blockade; ZIF-8, Zeolitic Imidazolate Framework-8; CaP, calcium phosphate; GSDME, gasdermin E.

### 2.2 Classification and construction of metal-based nanomaterials

The direct application of free metal ions is limited by poor targeting, high systemic toxicity, and rapid metabolism. Nanotechnology offers a diverse range of material platforms to overcome these obstacles. Based on their composition and structural characteristics, metal-based nanoplatforms are primarily classified into inorganic metal nanoparticles, MOFs, single-atom nanoenzymes, and organic nanocarriers doped or chelated with metal ions. Different types of materials have their advantages and disadvantages in terms of metal ion loading capacity, release behavior, biocompatibility, and immunomodulatory efficiency, and the selection must be tailored to the intended therapeutic mechanism.

Inorganic metal nanoparticles, such as iron oxide nanoparticles, manganese oxide nanoparticles, and gold nanoparticles, represent the most extensively studied class. Their preparation processes are relatively simple, and they exhibit high physicochemical stability. Some of these materials have already received clinical approval or are currently undergoing clinical trials. Iron oxide nanoparticles have been approved by the U.S. Food and Drug Administration (FDA) as magnetic resonance imaging (MRI) contrast agents. Their immunotherapeutic applications primarily rely on the ferroptosis effect induced by iron ions (Yang et al., 2025). Manganese oxide nanoparticles, in contrast, can degrade under the acidic conditions of the TME, releasing manganese ions *in situ* to activate the STING pathway (Fan Y. et al., 2025). The advantage of these materials lies in their dual-function synergy, wherein the nanoparticles serve both as carriers and as sources of immunomodulators; a drawback is that the release of metal ions typically lacks precise kinetic control and often depends on the material’s intrinsic degradation rate. MOFs are formed through the self-assembly of metal ions or clusters with organic ligands via coordination bonds. They possess extremely high specific surface areas and porosity, enabling efficient loading of metal ions and small-molecule drugs (Khulood et al., 2025). The structural tunability of MOFs allows for precise control over the coordination environment and release behavior of metal ions. For example, gallium-based MOFs co-loaded with manganese ions degrade and release gallium and manganese ions in the acidic TME, inducing mitochondrial-mediated apoptosis and cGAS-STING pathway activation, respectively, thereby achieving a cascade of apoptotic and immune responses (Ma et al., 2026). Potential limitations of MOFs include the need for further evaluation of the long-term biosafety of organic ligands, and the limited stability of some MOFs under physiological conditions. Single-atom nanoenzymes are a novel class of materials in which a single metal atom is dispersed and anchored on a solid support, combining the catalytic activity of natural enzymes with the design flexibility of nanomaterials (Yu et al., 2026). By mimicking the active sites of metalloenzymes, single-atom nanoenzymes can catalyze the generation of ROS, such as hydroxyl radicals, within the TME, thereby inducing ICD while avoiding systemic exposure to free metal ions. Compared to conventional nanoparticles, single-atom nanoenzymes exhibit higher catalytic efficiency with nearly 100% metal atom utilization; however, their preparation processes are complex, and scaling up production remains challenging. Organic nanocarriers doped or chelated with metal ions integrate metal ions into organic carriers such as polymer nanoparticles and liposomes through coordination or encapsulation. Such systems combine the excellent biocompatibility of organic carriers with the immunomodulatory functions of metal ions and can achieve active targeting or environment-responsive release through surface modification. For example, pH-responsive liposomes loaded with gallium and manganese ions and functionalized with neutrophil membranes can achieve tumor-targeted co-delivery of the two metal ions and prolong their circulation time *in vivo* (Ma et al., 2026). However, the metal-loading capacity of organic carriers is typically lower than that of inorganic materials, and the release of metal ions may be constrained by the degradation rate of the polymer.

**FIGURE 2:**
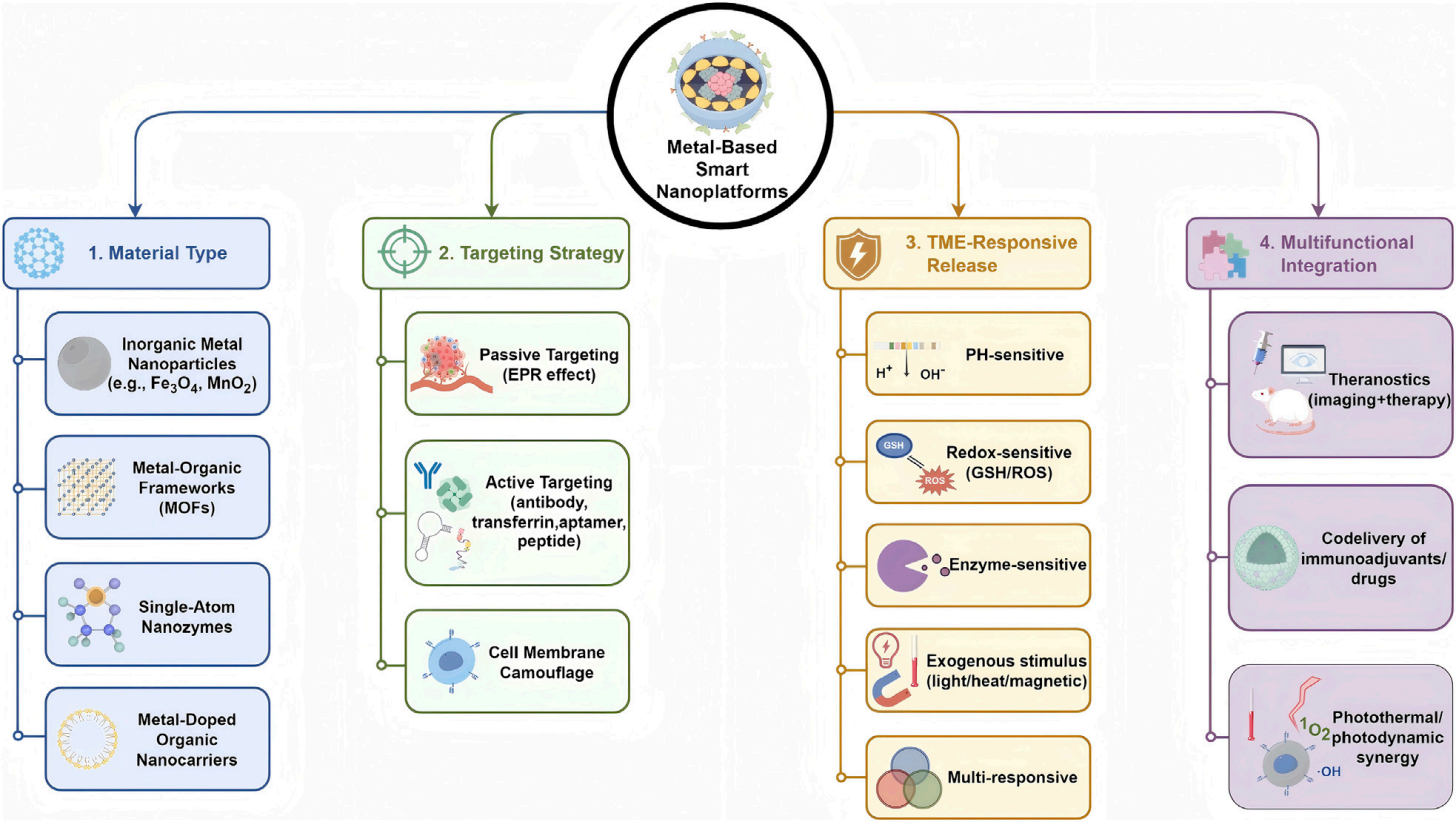
Design Strategies of Metal-Based Smart Nanodelivery Systems for Cancer Immunotherapy. This hierarchical diagram outlines four design branches centered on “Metal-Based Smart Nanoplatforms”: (1) Material Type (inorganic nanoparticles, MOFs, single-atom nanozymes, organic nanocarriers); (2) Targeting Strategy (passive EPR effect, active targeting via ligands, cell membrane camouflage); (3) TME-Responsive Release (pH, redox, enzyme, exogenous stimuli, multi-responsive); and (4) Multifunctional Integration (theranostics, codelivery, PTT/PDT synergy). Abbreviations: EPR, enhanced permeability and retention; GSH, glutathione; MOF, metal-organic framework; TME, tumor microenvironment.

Furthermore, the overall design strategy for metal-based smart nanodelivery systems comprises four core dimensions, as shown in Figure 2. First, the selection of material types includes inorganic metal nanoparticles such as iron oxide and manganese oxide, MOFs, single-atom nanoenzymes, and metal-ion-doped organic nanocarriers. Different materials have their advantages and disadvantages in terms of loading capacity and release behavior. Second, targeting strategies include passive targeting via the EPR effect or active targeting through antibodies, transferrin, aptamers, peptides, and biomimetic cell membrane camouflage. Third, TME-responsive release can be designed to be pH-sensitive, redox-sensitive (such as GSH-responsive or ROS-responsive), responsive to exogenous physical signals such as photothermal or magnetic stimuli, or a combination of multiple responses. Fourth, multifunctional integration combines PTT, PDT, and immunotherapy, or co-delivers immunostimulants and small-molecule drugs. These strategies provide a systematic framework for subsequent discussions on targeted delivery and responsive release. Table 2 implements this framework by matching each metal ion with its optimal nanoplatform based on immune targets and release kinetics.

**TABLE 2:** Mechanism–design matching framework for metal-based nanodrugs in cancer immunotherapy.

Metal ion	Primary immune target/ pathway	Required release kinetics	Rationale	Recommended nanoplatforms	References
Fe^2+^/Fe^3+^	Ferroptosis → ICD	Burst release (within hours)	Immunogenicity of ferroptosis diminishes rapidly	Hollow Fe/Fe_3_O_4_, Fe-based MOFs	Wei et al. (2025)
Mn^2+^	cGAS-STING direct agonist	Sustained or pH-triggered	Avoids chronic STING activation and T-cell exhaustion	MnO_2_ nanoparticles, Mn-ZIF-8	Li X. et al. (2025)
Cu^+^/Cu^2+^	Cuproptosis + PD-L1 upregulation	Efficient release with ICB co-delivery	PD-L1 upregulation may counteract immune activation	Cu-MOFs, copper-peptide nanoparticles	Luo et al. (2026)
Zn^2+^	TAM polarization, T-cell metabolism	Sustained release *in vivo* or *ex vivo* pulse	Prolonged exposure risks T-cell apoptosis	ZIF-8, Zn-doped nanoparticles	Mu et al. (2025)

Abbreviations: ICD, immunogenic cell death; cGAS, cyclic GMP-AMP, synthase; STING, stimulator of interferon genes; PD-L1, programmed death-ligand 1; ICB, immune checkpoint blockade; TAM, tumor-associated macrophage; MOFs, metal-organic frameworks; ZIF-8, Zeolitic Imidazolate Framework-8; MnO_2_, manganese dioxide.

### 2.3 Tumor-targeting delivery strategies

Achieving tumor-selective accumulation of metal ions is a prerequisite for reducing systemic toxicity and enhancing therapeutic efficacy. Current mainstream strategies include passive targeting and active targeting, and their combined optimization is required. Passive targeting primarily relies on the EPR effect of tumor tissue, allowing nanoparticles with particle sizes ranging from 20 to 200 nm to accumulate preferentially at the tumor site (Fan S. et al., 2025). This strategy requires no additional modification and is applicable to a wide range of tumors; however, the heterogeneity of the EPR effect limits its reliability when used alone, owing to species differences and tumor type variability (Belyaev et al., 2025). Active targeting further enhances enrichment and internalization efficiency by modifying the nanoparticle surface with ligands that can specifically bind to tumor cells or components of the TME. Commonly used ligands include antibodies (e.g., trimetallic nanozymes modified with anti-CD44 antibodies), transferrin (e.g., transferrin-modified gold nanostars that enhance uptake by breast cancer cells), and aptamers and peptides, which have low immunogenicity and synthesis costs. Cell membrane-mimicking camouflage strategies that have emerged, such as nanoplatforms coated with neutrophil membranes, can leverage the high expression of β1 integrin on the membrane surface to target vascular cell adhesion molecule-1 (VCAM-1) on tumor vascular endothelial cells, while simultaneously prolonging circulation time *in vivo* (Hameed et al., 2025). Active targeting can significantly improve tumor accumulation and internalization of nanoparticles, but ligand selection, coupling processes, and potential immunogenicity require careful optimization. In practical applications, passive and active targeting work synergistically and complement each other: initial tumor tissue enrichment is achieved through the EPR effect, followed by cell-specific recognition and internalization via active targeting. Characteristics of the TME, such as low pH and high GSH levels, can also be integrated with targeting strategies to construct multi-modal intelligent recognition systems.

### 2.4 Tumor microenvironment-responsive release

Responsive nanosystems can respond to pathological features specific to the TME and enable on-demand controlled release of metal ions, thereby improving treatment precision and reducing systemic toxicity (Wang Y. et al., 2024). Based on the source of the triggering signal, these responses can be classified into endogenous responses, including pH, GSH, and enzymes, and exogenous responses, including light, magnetic, and thermal stimuli. Different response mechanisms are suited for distinct applications and can be integrated into a single nanoplatform to achieve multi-modal intelligent regulation. Among endogenous responses, pH-responsive systems are the most common. Tumor tissues exhibit a slightly acidic environment due to enhanced glycolysis, with extracellular pH around 6.5–6.8 and endosomal or lysosomal pH as low as 4.5–5.5. pH-sensitive liposomes encapsulating gallium-based MOFs loaded with Mn^2+^ undergo membrane conformational changes in acidic environments, promoting fusion between the nanoparticles and tumor cells (Ma et al., 2026). A doxorubicin-gold nanoparticle system linked via an imidazole bond can also cleave and release the drug under acidic conditions (Dockery and Daniel, 2023). Redox-responsive systems utilize high intracellular GSH concentrations (2–10 mM) in tumor cells to reduce and cleave disulfide bonds and other responsive groups (Dockery and Daniel, 2023). For example, the trimetallic composite nanoenzyme CD44FMNA can release nitric oxide and generate hydroxyl radicals under GSH regulation (Wang X. et al., 2024). Enzyme-responsive systems utilize matrix metalloproteinases and cathepsins, which are specifically overexpressed in the TME, to trigger carrier degradation; however, their universality is limited by the heterogeneity of enzyme expression profiles across tumors. Exogenous responses achieve precise spatiotemporal control through physical signals such as light, heat, and magnetism and are not affected by tumor heterogeneity. Furthermore, the near-infrared band II (NIR-II) light-activated cobalt (III) nanomedicine NanoCo generates a photothermal effect under 1064 nm laser irradiation (64.2% efficiency) and accelerates the reduction of non-cytotoxic Co(III) to cytotoxic Co(II), achieving photothermal-chemical-immune synergistic therapy (Tang et al., 2026). The limitations of exogenous responses lie in the need for additional equipment and the limited tissue penetration depth of light. Multi-responsive systems integrate the aforementioned mechanisms; for example, a nanoplatform encapsulated by neutrophil membranes simultaneously possesses pH-responsive release and microenvironment-targeting capabilities (Ma et al., 2026), enabling graded responses based on signal intensity to enhance drug delivery precision, though this also increases design complexity. The selection of different response mechanisms must align with the required kinetics of metal ion release. Ions requiring rapid, burst-like release, such as Fe^2+^, are suitable for endogenous pH or redox responses, whereas those requiring precise spatiotemporal control favor exogenous light-triggered responses.

## 3 Application of metal nanomedicines in combination therapy

Through multiple mechanisms, including reshaping the immunosuppressive microenvironment, inducing ICD, and activating innate immune signaling pathways, metal nanomedicines achieve a greater-than-additive antitumor effect.

### 3.1 Combination with immune checkpoint blockade

Although ICIs have achieved clinical breakthroughs in various solid tumors, their efficacy is limited by insufficient immune cell infiltration in the TME, a condition known as the cold tumor state (Ouyang et al., 2025). Metal nanomedicines can convert cold tumors into hot tumors by inducing ICD or directly activating the STING pathway, thereby breaking through the barrier of ICI resistance (Wang L. et al., 2025). This conversion mechanism has been validated across multiple nanotechnology platforms. As an example, ZIF-8 nanoparticles degrade in the acidic TME to release zinc ions and co-loaded PD-L1 small interfering RNA (siRNA). Zinc ions trigger pyroptosis by inducing intracellular osmotic imbalance and a ROS burst; the released danger signals promote DC maturation and cytotoxic T-cell infiltration. Concurrently, PD-L1 siRNA downregulates PD-L1 expression on the tumor cell surface, relieving immune evasion and enhancing the efficacy of ICIs (Gong et al., 2025). Another strategy leverages the ability of cobalt ions to activate the cGAS-STING pathway. Hu et al. (Hu et al., 2026) constructed pH-responsive nanoclusters co-loaded with cobalt ions and lenvatinib; the former induces DNA damage and ICD, while the latter downregulates PD-L1 translation by inhibiting the MNK1–eIF4E pathway and promotes vascular normalization. When combined with anti-PD-1 antibodies, this nanocluster induced enhanced DC maturation, increased CD8^+^ T-cell infiltration, and a reduction in regulatory T-cell numbers in a mouse liver cancer model, demonstrating superior antitumor efficacy compared to monotherapy. These studies indicate that the synergy between metal nanomedicines and ICIs not only relies on antigen release from ICD but also alleviates immune suppression at multiple levels by actively regulating PD-L1 expression and the tumor vascular microenvironment.

### 3.2 Combination with photothermal/photodynamic therapy

PTT utilizes photothermal agents to convert light energy into thermal energy under near-infrared irradiation, directly inducing tumor cell necrosis; however, the danger signals released during this process can also stimulate an antitumor immune response (Han et al., 2025). Metal nanomaterials inherently possess both excellent photothermal conversion properties and immunomodulatory functions; the combination of these two capabilities generates a positive feedback loop in which thermotherapy enhances immunotherapy, and immunotherapy amplifies thermotherapy (Xie et al., 2025). NanoCo, a NIR-II light-activated Co(III) nanomedicine developed by Tang et al., is a typical example of this synergistic mechanism (Tang et al., 2026). Under 1064 nm laser irradiation, this nanomedicine achieves a photothermal conversion efficiency as high as 64.2%. It not only effectively generates heat to kill tumors but also accelerates the reduction of non-toxic Co(III) to cytotoxic Co(II), achieving spatiotemporal coupling of chemotherapy and PTT. More importantly, this process simultaneously induces ICD, promotes DC maturation, and activates T-cell responses. In 4T1 tumor-bearing mice, treatment with NanoCo combined with platinum-based drugs encapsulated in human serum albumin and light irradiation not only achieved complete tumor regression but also induced long-term immune memory, preventing tumor recurrence (Tang et al., 2026). Another strategy utilizes Lacto-LM, a gallium-based liquid metal nanocomposite engineered with lactic acid bacteria, where bacteria-derived pathogen-associated molecular patterns (PAMPs) serve as intrinsic immunostimulants. This synergistically enhances antitumor immunity with photothermal effects; a single intravenous injection combined with two laser irradiations can achieve regression of the primary tumor and inhibit metastasis (Sang et al., 2025). These studies suggest that the photothermal properties of metal nanomaterials and the immunomodulatory functions of metal ions possess inherent synergistic advantages. Coupling the two within a single platform can simultaneously achieve local tumor ablation and systemic immune activation.

The combination of bacteria and metal-based nanomaterials has recently attracted increasing attention as an emerging therapeutic strategy for cancer immunotherapy (Xiao et al., 2024). Bacteria-nanomaterial hybrids offer several unique advantages: bacteria provide natural tumor targeting, immune activation, and programmable behavior, while metal nanomaterials contribute photothermal conversion, catalytic activity, and controlled drug release (Xiao et al., 2024). Representative examples include iridium (III) photosensitizer-bacteria hybrids that activate both innate and adaptive immunity (Lin et al., 2023), bismuth-functionalized probiotics that combine Bifidobacterium infantis with bismuth-based nanoparticles for enhanced radiotherapy and immune activation (Xiao et al., 2025), and copper-based nanodrug-bacteria biohybrids (LGG-PDA@Cu-CPT) that stimulate DC maturation and T-cell activation (Zhou et al., 2026). Additionally, engineered bacterium-metal-organic framework biohybrids, such as SO@Hf-MOF-Pt, have been designed to reshape the radiosuppressive TME for enhanced radiotherapy (Wang J. W. et al., 2025). These studies collectively demonstrate that bacteria-metal nanomaterial hybrids represent a versatile and powerful platform for next-generation cancer immunotherapy, with the Lacto-LM system discussed above serving as a notable example within this emerging paradigm. The interplay between intratumoral microbiota and tumor immunosurveillance further underscores the therapeutic potential of bacteria-based strategies (Mai et al., 2025).

### 3.3 Combination with radiotherapy and chemotherapy

Radiotherapy and chemotherapy induce tumor cell death by directly damaging DNA, but they often fail to achieve complete tumor eradication due to dose-limiting toxicity and insufficient immune activation. Metal nanomaterials can serve as radiosensitizers or carriers for chemotherapeutic drugs, coupling local treatment with an immune response (Sen et al., 2022). The X-ray-responsive manganese-gold bimetallic nanoassembly (designated sMnAu NAs, small manganese-gold nanoassemblies) designed by Chen R. et al. (2025) demonstrates a typical approach for this strategy. This assembly cross-links approximately 140-nm MnAu nanoparticles into a nanoassembly via a diselenide-containing crosslinker, enabling efficient accumulation within tumors via the EPR effect; upon X-ray irradiation, the diselenide bonds break, causing the assembly to dissociate into small particles of approximately 6 nm, thereby achieving deep tumor penetration. The dissociated MnAu alloy nanoparticles provide localized energy deposition enhanced by gold for radiotherapy, while also controllably releasing Mn^0^, which rapidly converts to Mn^2+^ in the weakly acidic TME. The dual role of Mn^2+^ is particularly critical: it enhances ROS generation via a Fenton-like reaction, exacerbating oxidative damage to tumor cells; simultaneously, it activates the cGAS-STING pathway, converting radiation-induced DNA damage into a sustained innate immune signal. This triple synergistic strategy combining chemodynamic therapy, radiotherapy, and immunotherapy not only inhibits *in situ* tumor growth but also exerts systemic immune control on metastatic lesions (Chen R. et al., 2025). This study demonstrates that metal nanomaterials can transform traditional chemoradiotherapy from a purely local therapy into a local-systemic integrated immunotherapy regimen, with the STING-activating function of metal ions serving as the core driving force behind this transformation.

### 3.4 Combination with immune cell therapy

Immunotherapy, such as chimeric antigen receptor T-cell (CAR-T) therapy, has achieved significant success in hematologic malignancies; however, its efficacy in solid tumors is limited by T-cell functional exhaustion and immune suppression in the TME. Metal nanomaterials offer an alternative regulatory approach to genetic engineering. By briefly modulating the metal ion homeostasis within T cells through *ex vivo* pretreatment, they can enhance effector function without altering receptor specificity (Long et al., 2024). Gong et al. (2026) used zinc nanoparticles to briefly pre-stimulate T cells *ex vivo*, causing a transient increase in intracellular Zn^2+^ concentration. This treatment, which does not involve gene editing, induces mitochondrial metabolic remodeling and signaling pathway re-regulation via Zn^2+^, thereby enhancing the expression of genes associated with T cell effector function at the transcriptomic level without inducing oxidative stress or apoptosis. Pre-treated T cells demonstrated enhanced tumor-killing ability and secreted higher levels of IL-2, IFN-γ, and TNF-α at various effector-to-target ratios. In a mouse melanoma model, the transfer of pre-treated T cells delayed tumor growth and prolonged survival; when combined with a galectin-9 inhibitor, tumor control was further enhanced. In an xenograft model derived from ovarian cancer patients, pre-treated CAR-T cells similarly demonstrated superior tumor infiltration and control capabilities (Gong et al., 2026). The uniqueness of this strategy lies in its use of zinc ions as transient signaling molecules rather than persistent cytotoxic agents, “training” T cells within a short timeframe to acquire stronger stress adaptation and effector functions without altering their antigen-recognition receptors. This suggests that metal nanomaterials can serve not only as tumor-killing agents but also as functional reprogrammers of immune cells, opening new avenues for immunotherapy against solid tumors.

### 3.5 Summary of synergistic mechanisms

**FIGURE 3:**
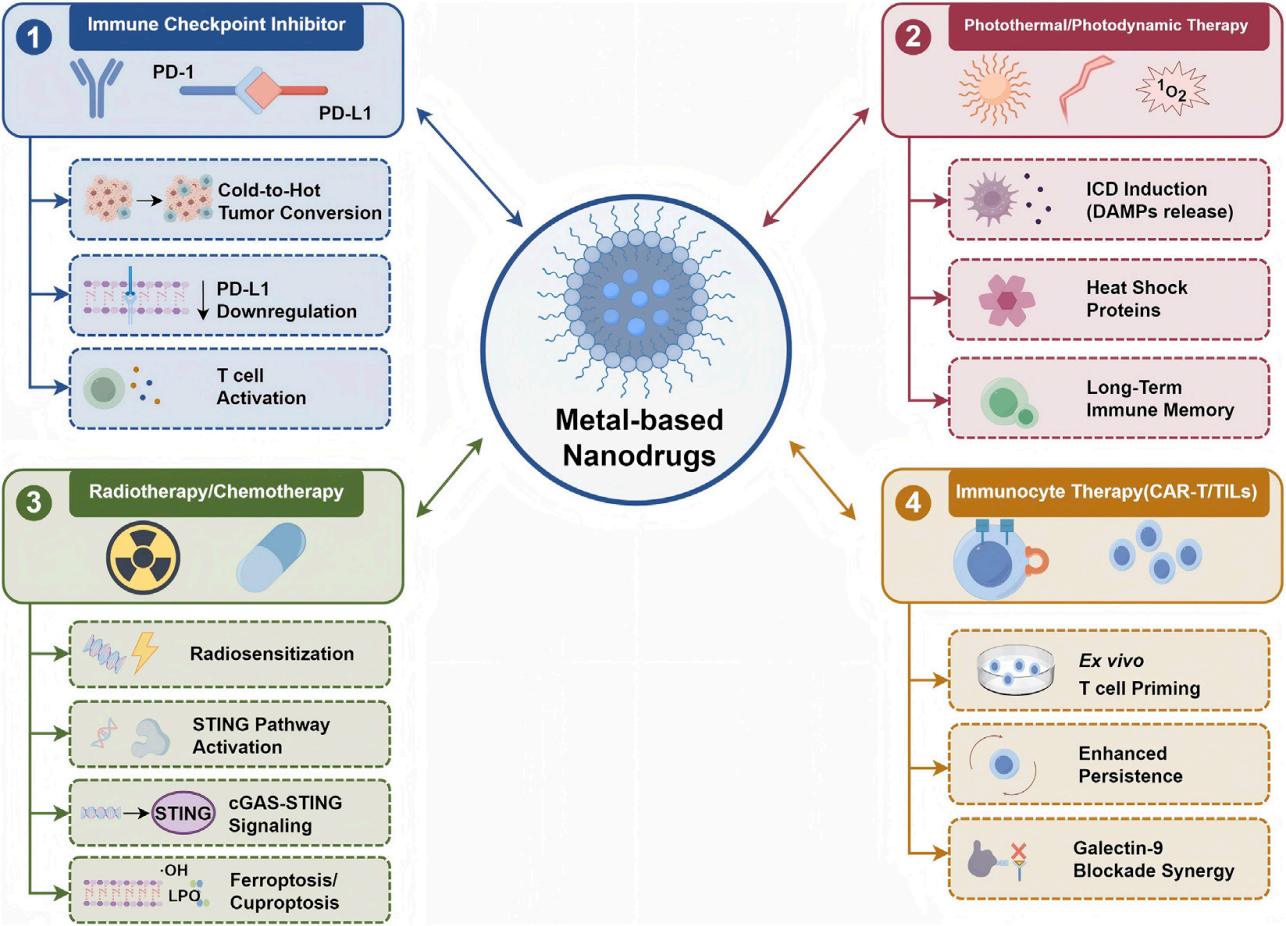
Synergistic Mechanisms of Metal-Based Nanodrugs in Cancer Immunotherapy. This hub-and-spoke diagram illustrates four combination therapy branches centered on “Metal-Based Nanodrugs”: (1) Immune Checkpoint Blockade, with sub-effects including cold-to-hot tumor conversion, PD-L1 downregulation, and T cell activation; (2) Photothermal/Photodynamic Therapy, contributing to ICD induction via DAMPs release and long-term immune memory; (3) Radiotherapy/Chemotherapy, achieving radiosensitization, STING pathway activation, and ferroptosis/cuproptosis; and (4) Immunocyte Therapy (CAR-T/TILs), involving *ex vivo* T cell priming and enhanced persistence. Arrows are labeled “Synergistic Effects” to emphasize cooperative antitumor efficacy. Abbreviations: CAR-T, chimeric antigen receptor T-cell; cGAS, cyclic GMP-AMP synthase; DAMP, damage-associated molecular pattern; ICD, immunogenic cell death; LPO, lipid peroxidation; PD-1, programmed cell death protein 1; PD-L1, programmed death-ligand 1; STING, stimulator of interferon genes; TILs, tumor-infiltrating lymphocytes.

Although the aforementioned combination therapy strategies target different aspects of immune evasion, the synergistic effects of metal nanomedicines can be attributed to three interrelated mechanisms of action (Figure 3). The first mechanism involves inducing ICD to release tumor antigens and danger signals, thereby activating DCs and initiating an adaptive immune response; this is particularly critical when combined with ICIs. The second mechanism involves directly manipulating innate immune signaling pathways, particularly the cGAS-STING axis. Metal ions such as manganese, cobalt, and zinc specifically activate this pathway, converting damage signals induced by local therapy into a sustained type I interferon response, thereby reshaping the immunological state of the TME. The third mechanism involves directly regulating the function and metabolism of immune cells themselves, such as the *in vitro* pretreatment of T cells with zinc nanoparticles, or the enhancement of antitumor capacity by modulating the polarization state of effector immune cells, such as TAMs. These three mechanisms are not mutually exclusive. Metal ion-induced ICD can further amplify the activation signals of the STING pathway, while STING pathway activation, in turn, promotes the cross-presentation of antigens from dying cells by DCs, forming a positive feedback loop. Consequently, metal nanomedicines can integrate multiple therapeutic modalities onto a single platform, including PTT, radiation therapy, chemotherapy, ICB, and immunotherapy, bridging the gap between local killing and systemic immunity to achieve enhanced synergistic antitumor efficacy.

## 4 Clinical translation prospects and challenges

### 4.1 Current status of clinical translation

To provide a clearer translational landscape, it is important to first distinguish between nanoplatforms whose primary therapeutic mechanism is immunomodulation and those in which immune activation is only a secondary or incidental effect. The latter category includes imaging contrast agents, drug delivery vehicles, and photothermal ablation platforms that were not originally designed for immunotherapy but have been observed to elicit certain immune responses. In this section, we focus primarily on metal-based nanoplatforms that have entered clinical development, while acknowledging that many of these were not specifically developed for immunotherapy. Metal-based immunotherapy is still in its early stages of development, but some metal nanomedicines have already entered clinical trials. Currently, the most rapid progress is concentrated in two categories: iron oxide nanoparticles and gold nanoparticles. The first category comprises clinically approved metal nanomaterials. Iron oxide nanoparticles are among the most clinically mature metal nanomedicines; thanks to their excellent biocompatibility and magnetic properties, they have already been applied in MRI, intravenous iron supplementation, and magnetothermy (Guo et al., 2025). Among these, the superparamagnetic iron oxide nanoparticle ferumoxytol has been approved by the FDA for the treatment of iron-deficiency anemia and can also serve as a drug carrier and MRI contrast agent (Yang et al., 2025). It should be noted, however, that ferumoxytol was not originally developed as an immunotherapeutic agent; its immunomodulatory properties, such as the ability to polarize tumor-associated macrophages toward the M1 phenotype, have been recognized more recently and are currently being explored in preclinical and early clinical settings. Clinical exploration of gold nanoparticles is also quite active. NU-0129 is a spherical nucleic acid-conjugated gold nanoparticle that has completed a Phase 0 trial in patients with recurrent glioblastoma. demonstrating tumor penetration and knockdown of the target gene Bcl2L12, though no objective response was observed (Kumthekar et al., 2021). CYT-6091 is a formulation of PEGylated gold nanoparticles coupled with recombinant human tumor necrosis factor. In a Phase I trial for advanced solid tumors, the maximum tolerated dose was not reached (maximum administered dose, 600 μg/m^2^); only 2 patients (7%) experienced Grade 3 hypotension, and no Grade 4 adverse events were reported; one patient with ocular melanoma achieved a partial response (PR), and four achieved stable disease (SD); the overall objective response rate was limited, highlighting the significant gap between preclinical synergistic effects and clinical efficacy (Libutti et al., 2007; Libutti et al., 2010). AuroShell is a silica-gold core-shell nanostructure used for photothermal ablation of head and neck tumors; preliminary studies have been completed, but trials for lung tumors were terminated due to slow patient enrollment (A Pilot Study of AuroLase(tm), 2009). The progress of clinical trials for the aforementioned gold nanoparticles is summarized in Table 3. Domestically, a nano-carbon iron suspension for injection, which uses Fe^2+^ as the active ingredient and nano-carbon as the carrier, has been approved by the National Medical Products Administration (NMPA) to conduct a Phase II clinical trial for solid tumors in combination with radiotherapy. The trial is currently progressing smoothly, and no similar products are on the market (Xie et al., 2020; Xie et al., 2023). For these platforms, it is important to recognize that their primary indications are not immunotherapy, and their immunomodulatory effects, while promising, remain secondary to their primary therapeutic functions. Although these studies demonstrate translational potential, the overall clinical success rate of nanomedicines remains low. According to Park et al. (Hu et al., 2025), only approximately 1%–5% of all nanomedicine development projects ultimately reach clinical application, with the majority failing during Phase II or III trials. This translation gap highlights the systemic challenges that metal nanomedicines face in transitioning from the laboratory to clinical practice. These observations collectively indicate that metal-based cancer immunotherapy remains at an early, exploratory stage, and the clinical maturity of this field should not be overstated.

**TABLE 3:** Gold nanomedicines in clinical trials.

Trial Identifier	Intervention name	Nanoplatform description	Therapeutic area	Indication	Phase	Status	Key results summary
NCT03020017	NU-0129	Spherical nucleic acid gold nanoparticle	Cancer therapy	Glioblastoma/ gliosarcoma	Phase 0	Completed	Tumor penetration; Bcl2L12 knockdown; no DLT; no objective responses
NCT00356980	CYT-6091	PEGylated colloidal gold nanoparticles conjugated with recombinant human TNF-α	Cancer therapy	Advanced solid tumors	Phase 1	Completed	No MTD reached (highest dose 600 μg/m^2^); grade 3 hypotension in 2/29 patients (7%), no grade 4; PR in 1 patient (ocular melanoma), SD in 4 patients; no DLT.
NCT00848042	AuroLase	PEGylated silica-gold nanoshell (AuroShell)	Photothermal ablation	Refractory/recurrent head and neck tumors	Pilot Study	Completed	No data available
NCT01679470	AuroLase	PEGylated silica-gold nanoshell (AuroShell)	Photothermal ablation	Primary or Metastatic Lung Tumors	N/A	Terminated	Terminated due to poor accrual; no efficacy data reported

Abbreviations: DLT, dose-limiting toxicity; MTD, maximum tolerated dose; AE, adverse event; SD, stable disease; PR, partial response; CR, complete response; N/A, not applicable. Data for NU-0129 and CYT-6091 are from published phase 0/I trials; the two AuroLase trials have not reported formal efficacy results.

### 4.2 Scalable preparation and quality control

The clinical translation of metal nanomedicines first requires addressing large-scale production. Industrial-scale production demands batch-to-batch reproducibility and stability while maintaining the key physicochemical properties of nanoparticles, such as size, morphology, surface charge, and crystalline phase (Sharma et al., 2024). Currently, various methods for large-scale preparation have been developed, including microemulsion synthesis, resonant acoustic mixing, microfluidic technology, and supercritical fluid synthesis. Among these, microfluidic technology enables continuous flow preparation and excellent process control, making it suitable for nanoparticle formulations with strict particle size distribution requirements; resonant acoustic mixing is suitable for high-viscosity systems and can avoid shear damage caused by traditional stirring blades (Shahalaei et al., 2024). Key quality control parameters include particle size distribution, morphological uniformity, surface chemistry, metal content, colloidal stability, and sterility and endotoxin levels. Taking iron oxide nanoparticles as an example, size and morphology determine their pharmacokinetic characteristics and the extent of uptake by the reticuloendothelial system; surface modification influences protein corona formation and *in vivo* distribution; while crystal structure and magnetic anisotropy determine magnetic susceptibility and MRI contrast efficiency (Guo et al., 2025). The application of process analytical technologies (PAT) is key to achieving real-time quality control, enabling online monitoring of parameters such as particle size and concentration during production to promptly detect batch-to-batch variations. Future efforts should focus on further optimizing continuous-flow preparation processes, advancing real-time monitoring technologies, and developing systematic material characterization methods to facilitate the transition of metal nanomedicines from laboratory samples to industrial-scale products.

### 4.3 Biosafety assessment

The interactions between metal nanoparticles and biological systems are complex, and their toxicological profiles are influenced by various factors, including particle size, shape, surface charge, and coatings (Mehmood et al., 2025). Studies have shown that particles smaller than 10 nm exhibit higher genotoxicity, and the cytotoxicity of cationically modified particles can be two to three times that of anionic or neutral surfaces. Toxicological effects primarily include dose-dependent cytotoxicity, hepatic accumulation (accounting for approximately 30%–40% of the injected dose), oxidative stress, and immune activation (Mehmood et al., 2025). Taking iron oxide nanoparticles as an example, iron ions released following their degradation in lysosomes enter the body’s iron metabolism network, potentially disrupting normal iron homeostasis (Guo et al., 2025). To improve safety, researchers have developed various strategies. PEGylation (polyethylene glycol modification) is a widely established strategy to reduce macrophage uptake and prolong circulation time; biodegradable hybrid systems can reduce long-term organ accumulation by 70%–80%; and responsive controlled-release systems can reduce the dosage required for effective treatment by 30%–50% (Mehmood et al., 2025). Furthermore, computational toxicology tools, such as machine learning models, have achieved a toxicity prediction accuracy of approximately 86.1%, aiding in the screening of low-risk candidate materials during the preclinical stage and accelerating the safety evaluation process (Ha et al., 2025).

### 4.4 Regulatory science pathways

The unique characteristics of metal nanomedicines pose new challenges to existing regulatory frameworks. Regulatory agencies such as the U.S. FDA, the European Medicines Agency (EMA), and China’s NMPA have successively issued guidance documents on nanomedicines, providing a fundamental basis for product development and review (Rodríguez-Gómez et al., 2025). However, the degradation products of metal nanoparticles, specifically free metal ions, may interfere with the body’s metal homeostasis network; their immunomodulatory functions, while forming the basis of therapeutic efficacy, may also pose safety risks. Therefore, the existing regulatory framework requires the establishment of specific evaluation criteria for metal nanomedicines, including clarifying the *in vivo* metabolic pathways and sites of accumulation of metal elements, assessing the long-term impact of degradation products on the body’s metal homeostasis, and establishing risk-benefit balance criteria between immunomodulatory effects and immunotoxicity. Given the significant differences in the physicochemical properties and biological activities of various metal elements, a uniform regulatory strategy is impractical; it is necessary to develop a tiered and categorized review system based on material classification and mechanisms of action. Early communication between regulatory authorities and developers, such as the FDA’s PRE-submission program or the NMPA’s communication system, is crucial for clarifying data requirements and accelerating product translation (Desai et al., 2025). The successful clinical translation of metal nanomedicines requires not only systematic and comparable safety data from basic researchers but also concurrent advancements in regulatory science to establish clear and feasible review pathways for this emerging therapy.

### 4.5 Challenges and perspectives

Although metal-based nanomedicines show great promise in tumor immunotherapy, their clinical translation still faces multiple significant limitations. Moderate activation of the cGAS-STING pathway is beneficial for antitumor immunity, whereas excessive or sustained activation may have adverse effects, such as upregulating CD73 to promote adenosine production, inducing the expansion of regulatory T cells (Tregs), or even directly inhibiting T-cell effector function (Fu et al., 2024). In tumors with pre-existing chromosomal instability, chronic STING signaling may even promote tumor progression and epithelial-mesenchymal transition (EMT). This necessitates that metal nanodrugs be designed with TME-responsive release mechanisms to achieve moderate, transient activation of STING signaling, thereby avoiding exceeding the upper limit of the safety window (Li et al., 2023). Different metal ions follow distinct metabolic pathways, leading to varying organ-specific toxicities. Manganese ions exhibit neurotoxicity; long-term accumulation can induce Parkinson’s disease. Copper ions primarily damage the liver; excessive accumulation can lead to hepatocyte death and fibrosis. Iron ions induce oxidative stress via the Fenton reaction, causing iron-overload-related cardiac and hepatic damage (Fontes et al., 2024). Furthermore, the body possesses a sophisticated homeostatic regulatory network for metal ions; the introduction of exogenous metal ions activates endogenous compensatory mechanisms, significantly diminishing the actual therapeutic efficacy of nanomedicines. Existing studies indicate that iron-based nanoparticles can upregulate ferritin expression, while zinc-based MOFs can induce the upregulation of metallothioneins. The functional impact of ferritin upregulation has been directly demonstrated: Zhao et al. (2025) showed that intracellular free iron is maintained at low concentrations due to sequestration in ferritin and efflux via ferroportin, significantly limiting the efficacy of iron-based ferroptosis therapy. However, systematic research on how to circumvent these compensatory mechanisms remains lacking. To overcome this obstacle, intermittent dosing can be employed to avoid continuous activation of compensatory mechanisms; combination with homeostasis-regulating drugs such as ferritin antagonists or copper chelators can be considered; or, through TME-targeted controlled release and protein coat regulation, systemic exposure can be reduced and the release rate of metal ions slowed (Chen et al., 2026).

In the long term, precision metal immunotherapy requires the integration of single-cell sequencing, spatial transcriptomics, and metallomics technologies to elucidate the sensitivity of metal ions and the characteristics of the immune microenvironment across different tumor types. By incorporating artificial intelligence algorithms, models can be established to predict the relationship between metal ions, immune phenotypes, and treatment outcomes, thereby enabling patient stratification and personalized therapy. Next-generation smart response systems should establish a nanotechnology platform capable of coordinating multiple responses, enabling precise identification and closed-loop feedback regulation of complex signals in the TME, such as pH, GSH, ROS, enzymes, and hypoxia. Concurrently, these systems should explore combined triggering mechanisms involving exogenous physical signals such as light, magnetic fields, and ultrasound as well as endogenous chemical signals, while prioritizing the use of clinically approved biodegradable materials to mitigate safety risks. Regarding multimodal synergistic strategies, in addition to continuing to optimize combination applications with ICIs and photothermal or radiotherapy, the synergistic potential of metal nanomedicines with mRNA vaccines, oncolytic viruses, and epigenetic drugs should also be explored. Furthermore, the inherent imaging and tracing capabilities of metal nanoparticles can endow them with integrated diagnostic and therapeutic capabilities. The development of a translation-oriented process and evaluation system requires the establishment of continuous-flow manufacturing processes and real-time quality control technologies, the development of high-throughput standardized safety evaluation methods such as organ-on-a-chip and microphysiological systems, and the creation of a biosafety database covering different material types and long-term observation periods. At the regulatory science level, efforts should be made to promote international regulatory coordination to unify the definitions, classifications, and evaluation standards for metal nanomedicines, encourage early regulatory communication, and establish specific review guidelines addressing the metabolism and long-term toxicity of metal elements. Although clinical translation still faces multiple challenges, through the synergistic advancement of basic research, translational medicine, and regulatory science, metal nanomedicines are expected to make the leap from the laboratory to the clinic on a path characterized by precision, intelligence, and safety.

## 5 Conclusion

This review systematically summarizes research progress on metal nanomedicines in tumor immunotherapy, focusing on the multi-target network through which metal ions such as manganese, iron, copper, and zinc reshape the TME by activating the cGAS-STING pathway, inducing ICD, and regulating immune cell function. Smart nanodelivery systems enable precise spatiotemporal control of metal ions, transforming metal nanomedicines from mere carriers into ideal platforms for combination therapy. In combination with ICB, PTT or PDT, radiotherapy, and immunotherapy, metal nanomedicines demonstrate unique synergistic advantages. Although iron oxide nanoparticles and gold nanoparticles have entered clinical trials, clinical translation still faces multiple challenges, including large-scale production, biosafety assessment, and regulatory science, with an overall success rate of less than 5%. By precisely engineering the biological functions of metal ions and combining patient stratification with smart response design, metal nanomedicines are expected to expand the population benefiting from immunotherapy from patients with hot tumors to those with cold tumors, becoming an important direction for next-generation precision immunotherapy.

## Glossary

**CAR** **-T**: chimeric antigen receptor T-cell
cGAMP: cyclic GMP-AMP
cGAS: cyclic GMP-AMP synthase
DC: dendritic cell
DLAT: dihydrolipoamide S-acetyltransferase
EMA: European Medicines Agency
EMT: epithelial-mesenchymal transition
EPR: enhanced permeability and retention
FDA: Food and Drug Administration
GSH: glutathione
HMGB1: high mobility group box 1
ICB: immune checkpoint blockade
ICD: immunogenic cell death
ICI: immune checkpoint inhibitor
MOF: metal-organic framework
MRI: magnetic resonance imaging
mtDNA: mitochondrial DNA
NIR-II: near-infrared region II
NMPA: National Medical Products Administration
PAMPs: pathogen-associated molecular patterns
PAT: process analytical technology
PD-L1: programmed death-ligand 1
PDT: photodynamic therapy
PR: partial response
PTT: photothermal therapy
ROS: reactive oxygen species
SD: stable disease
siRNA: small interfering RNA
STING: stimulator of interferon genes
TAM: tumor-associated macrophage
TCA: tricarboxylic acid
TME: tumor microenvironment
Treg: regulatory T cell
VCAM-1: vascular cell adhesion molecule-1
